# Complete chloroplast genome features and phylogenetic analysis of *Eruca sativa* (Brassicaceae)

**DOI:** 10.1371/journal.pone.0248556

**Published:** 2021-03-12

**Authors:** Bin Zhu, Fang Qian, Yunfeng Hou, Weicheng Yang, Mengxian Cai, Xiaoming Wu

**Affiliations:** 1 School of Life Sciences, Guizhou Normal University, Guiyang, China; 2 Oil Crops Research Institute, Chinese Academy of Agricultural Sciences, Wuhan, China; Huazhong University of Science and Technology, CHINA

## Abstract

*Eruca sativa* Mill. (Brassicaceae) is an important edible vegetable and a potential medicinal plant due to the antibacterial activity of its seed oil. Here, the complete chloroplast (cp) genome of *E*. *sativa* was *de novo* assembled with a combination of long PacBio reads and short Illumina reads. The *E*. *sativa* cp genome had a quadripartite structure that was 153,522 bp in size, consisting of one large single-copy region of 83,320 bp and one small single-copy region of 17,786 bp which were separated by two inverted repeat (IRa and IRb) regions of 26,208 bp. This complete cp genome harbored 113 unique genes: 79 protein-coding genes, 30 tRNA genes, and four rRNA genes. Forty-nine long repetitive sequences and 69 simple sequence repeats were identified in the *E*. *sativa* cp genome. A codon usage analysis of the *E*. *sativa* cp genome showed a bias toward codons ending in A/T. The *E*. *sativa* cp genome was similar in size, gene composition, and linearity of the structural region when compared with other Brassicaceae cp genomes. Moreover, the analysis of the synonymous (Ks) and non-synonymous (Ka) substitution rates demonstrated that protein-coding genes generally underwent purifying selection pressure, expect *ycf1*, *ycf2*, and *rps12*. A phylogenetic analysis determined that *E*. *sativa* is evolutionarily close to important *Brassica* species, indicating that it may be possible to transfer favorable *E*. *sativa* alleles into other *Brassica* species. Our results will be helpful to advance genetic improvement and breeding of *E*. *sativa*, and will provide valuable information for utilizing *E*. *sativa* as an important resource to improve other *Brassica* species.

## Introduction

*Eruca sativa* Mill. is an annual or perennial species of *Eruca* (Brassicaceae), mainly distributed in Europe and Western Asia. *E*. *sativa* is believed to have originated from the Mediterranean region and has been widely used as an oil crop and edible vegetable [1]. Due to the fragrance of its leaves, *E*. *sativa* is also a popular salad and spice in Middle Eastern and European countries [2]. Moreover, recent studies have demonstrated that *E*. *sativa* has many medicinal and therapeutic properties, including antioxidant and antimicrobial activities [3] as well as he ability to reduce neuroinflammation and testicular silver toxicity [4]. Additionally, *E*. *sativa* is resistant to white rust, drought, and aphids [5], traits that are urgently needed for cultivated *Brassica* species. Therefore, many attempts have been made to use somatic fusion and sexual hybridization to transfer such desirable agronomic traits from *E*. *sativa* to other *Brassica* species [6]. A phylogenetic analysis between *E*. *sativa* and other species in Brassicaceae family will undoubtedly improve the ability to transfer desirable agronomic traits to related species.

Chloroplasts (cp) have an independent circular genome and play essential roles in photosynthesis, development, and the physiology of green plants [7, 8]. Cp genomes generally have a quadripartite cyclic structure (120–160 kb in size), and harbor 110–130 unique genes [9]. The typical quadripartite cyclic structure of most angiosperms is comprised of a large single copy (LSC) region and a small single copy (SSC) region, which are divided by a pair of inverted repeats (IRa and IRb) [10, 11]. The evolutionary rate of the cp genome is much lower than that of the nuclear genome [12], due to fewer recombination incidents, lower nucleotide replacement rates, and the typical maternal inheritance of the cp genome. Therefore, the cp genome has been widely employed to decipher the genealogical relationships among species [13–16].

Many cp genomes have recently been decoded due to the advancements in next-generation sequencing technology, particularly third-generation sequencing technology which yields reads longer than 10 kb [16–21]. Thus, phylogenetic analyses based on cp genome data have become increasingly popular, even in small taxonomic groups [22–24]. Although a large number of cp genomes of Brassicaceae species have been sequenced, the plastid genome of *E*. *sativa* is not available.

In the present study, the complete cp genome of *E*. *sativa* was *de novo* assembled with a combination of long PacBio reads and short Illumina reads, and the features of this cp genome were fully elucidated. Next, 59 cp genomes of other species in the Brassicaceae family from GenBank were used to determine the genealogical relationships between *E*. *sativa* and other species. Our results will enable further genetic improvements and breeding of *E*. *sativa* and provide valuable information for utilizing *E*. *sativa* as a resource to improve of important *Brassica* species.

## Materials and methods

### Plant materials and cp DNA extraction

*E*. *sativa* seeds were provided by Professor Zaiyun Li (Huazhong Agriculture University), and cultivated in a glasshouse at Guizhou Normal University (Guiyang, China). A total of 5 g of fresh young *E*. *sativa* leaves of were collected to isolate cp DNA using the Plant DNA Extraction Mini Kit C (Onrew, Beijing) according to the manufacturer’s instructions. After determining the integrity of the DNA, 1 μg of DNA was fragmented, and a short-insert library (with the insertion of 450 bp) was constructed for Illumina sequencing (HiSeq X Ten), according to the manufacturer’s instructions (Illumina, USA). Then, 5 μg DNA was used to prepare the DNA libraries with insert sizes of 20 kb for PacBio sequencing, according to the manufacturer’s instructions (Pacific Bioscience Inc., Menlo Park, CA, USA). All the raw data, including short Illumina reads and long PacBio reads were submitted to the figshare (https://figshare.com) with the DOI: 10.6084/m9.figshare.13653515.

### Cp genome assembly and genes annotation

The 150 bp paired-end reads were produced by the Illumina sequencing platform. After removing the sequencing adapters and low-quality reads, the clean reads were obtained by Trimmomatic [25], according to the default options. To remove the nuclear reads, the clean reads were aligned to the published *Arabidopsis thaliana* (NC_000932) cp genome using BLASR (Basic Local Alignment with Successive Refinement) [26] (E-value: 10–6). Then, these selected short reads were used to assemble scaffolds using SOAPdenovo v2.04 according to the default parameters [27]. The low-quality PacBio reads (minimum read length of 500 bp and minimum read quality of 0.80) were removed from the raw data. The long selected PacBio reads were employed to fill the gaps within the scaffolds with PBJelly [28]. To correct possible mis-assemblies and errors, the Illumina reads were aligned to the assembled cp genome using BWA (version 0.5.9) with the default settings [29]. Frame-shift errors were manually corrected during gene prediction.

The Dual Organellar Genome Annotator was used to annotate the *E*. *sativa* cp genome with default settings [30]. The start and stop codons of each gene were verified by homology searches using BLAST (Basic Local Alignment Search Tool). Then, the *E*. *sativa* circular gene map was drawn in OGDraw software version 1.2 [31]. The well-annotated cp genome of *E*. *sativa* is available in the public GenBank database (https://www.ncbi.nlm.nih.gov/) under the accession number of MT013255.

### Repeat sequence analyses

The long repeat sequences of the *E*. *sativa* cp genome, including the palindrome, reverse, forward, and complement types, were detected by the web-service REPuter (https://bibiserv.cebitec.uni-bielefeld) [32] with the following settings: minimal repeat size, 30; sequence consistency, >90%; and maximum computed repeats to 50. Additionally, simple sequence repeat (SSR) loci were detected using MISA (https://webblast.ipk-gatersleben.de/misa/) [33] with the following settings: 10 repeats for mono-types, five repeats for di-types, four repeats for tri-, and three repeats for tetra-, penta- and hexa-types, respectively.

### Codon bias usage analysis

To understand the translation dynamics of the *E*. *sativa* cp genome, the CodonW1.4.2 program [34] (http://downloads.fyxm.net/CodonW-76666.html) was employed to calculate the synonymous codon usage of the protein-coding genes with default settings. The relative synonymous codon usage (RSCU) of all coding genes was also analyzed.

### Comparison of related cp genomes

The mVISTA program (http://genome.lbl.gov/vista/mvista/submit.shtml) [35] was used to analyze sequence divergence between the *E*. *sativa* cp genome and those of six related species. The related cp sequences were downloaded from the National Center for Biotechnology Information (NCBI), including *B*. *rapa* (NC_040849), *B*. *oleracea* (NC_O41167), *B*. *juncea* (NC_0282720), *B*. *nigra* (NC_030450), *B*. *napus* (NC_016734) and *A*. *thaliana* (NC_000932). IRscope (https://irscope.shinyapps.io/irapp/) was used to compare LSC/IRb/SSC/IRa junction regions among the seven selected cp genomes according to the annotated information.

### Analysis of the molecular evolution of coding genes

Pairwise comparisons of 79 protein-coding genes shared between *E*. *sativa* and six related Brassicaceae species were employed to calculate non-synonymous (Ka) and synonymous (Ks) substitution rates. Pairwise alignments of these genes were carried out by with MAFFT, and the Ka/Ks value was determined with KaKs calculator (version 2.0) according to the default parameters.

### Phylogenetic relationship analysis

To analyze the phylogenetic relationships between *E*. *sativa* and related Brassicaceae species, 59 Brassicaceae species cp genomes (S1 Table) were downloaded from GenBank to construct phylogenetic trees. In total, 61 homologous protein-coding sequences: *atpA*, *atpB*, *atpE*, *atpF*, *atpH*, *atpI*, *clpP*, *ndhA*, *ndhB*, *ndhC*, *ndhD*, *ndhE*, *ndhF*, *ndhG*, *ndhH*, *ndhI*, *ndhJ*, *petA*, *petB*, *petD*, *petG*, *psaA*, *psaB*, *psaC*, *psaI*, *psbA*, *psbB*, *psbC*, *psbD*, *psbE*, *psbF*, *psbH*, *psbI*, *psbK*, *psbL*, *psbM*, *psbN*, *psbT*, *rbcL*, *rpl2*, *rpl16*, *rpl20*, *rpl22*, *rpl23*, *rpl32*, *rpl33*, *rpl36*, *rpoA*, *rpoB*, *rpoC1*, *rpoC2*, *rps2*, *rps3*, *rps4*, *rps7*, *rps8*, *rps11*, *rps14*, *rps18*, and *ycf4*, among the selected Brassicaceae species cp genomes were used to determine the phylogenetic relationships according to the maximum likelihood (ML) method with 1000 replicates using MEGA7 [36].

## Results and discussion

### Cp genome assembly and features

**Fig 1:**
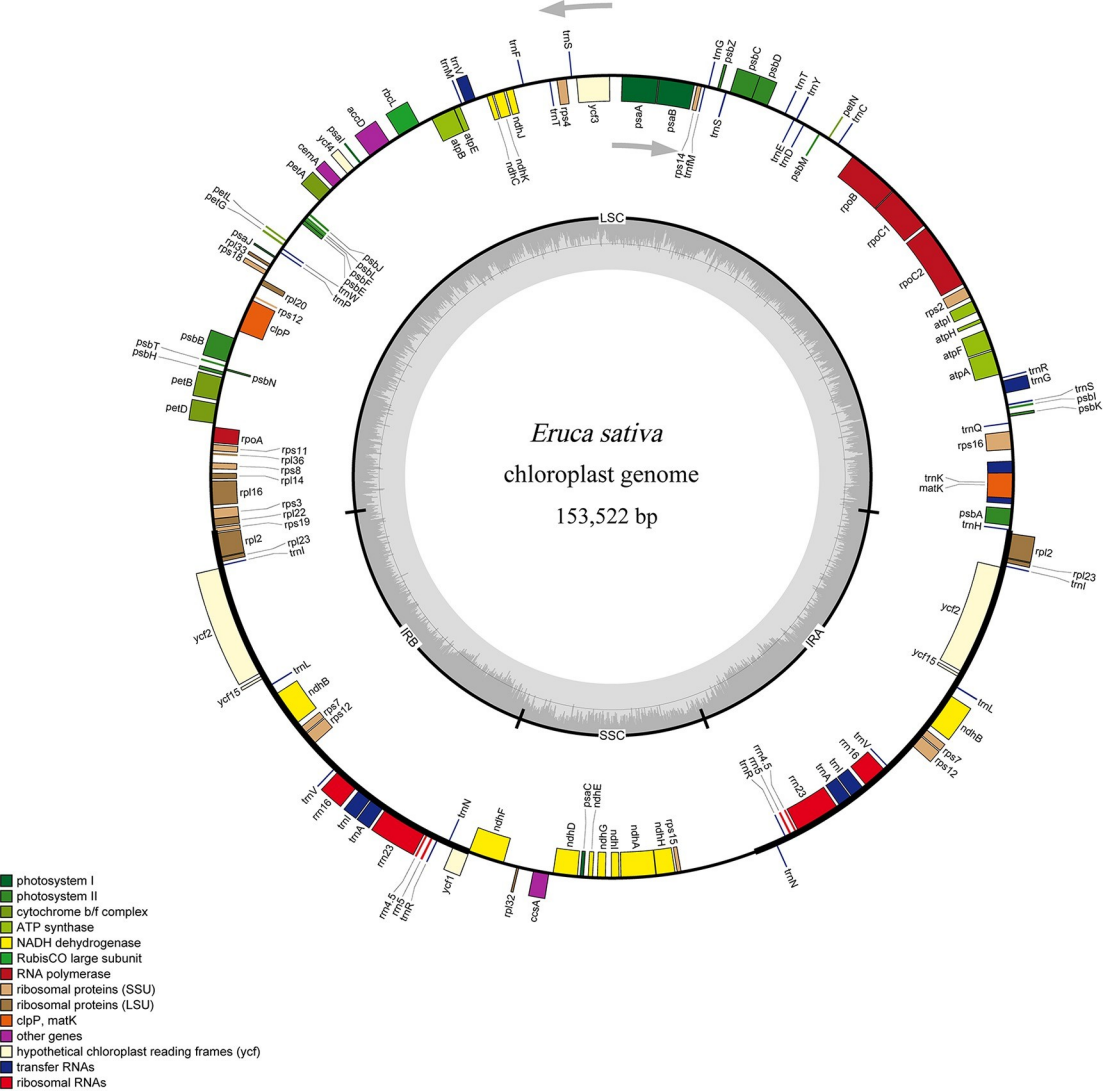
The circle gene map of the *E*. *sativa* cp genome. Genes drawn outside and inside of the circle are transcribed clockwise and counterclockwise, respectively. Genes belonging to different functional groups are color coded. The darker gray in the inner circle corresponds to GC content. SSC region, LSC region, and inverted repeats (IRA and IRB) are indicated.

The Illumina sequencing platform generated 8,078 Mb of raw data, resulting in an average coverage of more than 50,000 over the cp genome. After removing the adapters and low-quality reads, 7,722 Mb of clean data were obtained with an average Q20 of 97.62%. The PacBio platform generated 20,921 subreads with an N50 length of 4,698 bp and an average length of 4,002 bp (S2 Table). Both the Illumina reads and the PacBio subreads were used together to assemble the *E*. *sativa* cp genome (see Materials and Methods section). The complete *E*. *sativa* cp genome had a quadripartite structure comprised of 153,522 bp, including an SSC region of 17,786 bp and an LSC region of 83,320 bp, which were separated by a pair of inverted repeats (IRa and IRb) of 26,208 bp (Table 1, Fig 1). The average GC content of the cp genome was 36.38%, and the IR regions had the highest GC content (42.25%), followed by the LSC (34.15%), and SSC regions (29.23%). The *E*. *sativa* cp genome encoded 113 unique genes: 79 protein-coding genes, 30 tRNAs, and four rRNAs. This result is similar to previous findings on the whole cp genomes of *B*. *juncea* and *B*. *oleracea* [37]. The average gene length was 867 bp, and protein-coding gene regions accounted for 65.65% of the total sequence. The total length of the genic regions was 74,547 bp, representing 48.46% of the whole cp genome. A total of 82 genes, including 59 protein-coding genes and 23 tRNAs, were observed in the LSC regions. A total of 28 genes: five protein-coding genes, five tRNAs, and four rRNAs, were repeated in the IR regions, while only 11 genes were found in the SSC regions. Among the 113 genes, 14 genes (eight protein-coding genes and five tRNAs) harbored a single intron, whereas three genes (*rps12*, *clpP*, and *ycf3*) possessed two introns (Table 2). Moreover, *rps12* was a trans-spliced gene, as reported previously [38]. Detailed information about the gene copy number, the number of introns, and the gene functions are listed in Table 2.

**Table 1:** The detail characteristics of the complete cp genome of *E*. *sativa*.

Category	Items	Descriptions
Construction of cp genome	LSC region (bp)	83,320
	IRA region (bp)	26,208
	SSC region (bp)	17,786
	IRB region (bp)	26,208
	Genome Size (bp)	153,522
Gene content	Total genes (unique)	113
	Protein-coding genes	79
	tRNAs	30
	rRNAs	4
	Two copy genes	17
	Genes on LSC region (total)	82
	Genes on IRA region (total)	18
	Genes on SSC region (total)	11
	Genes on IRB region (total)	19
	Gene total length (bp)	74,547
	Average of genes length (bp)	867
	Gene length / Genome (%)	48.56
GC content	GC content of LSC region (%)	34.15
	GC content of IRA region (%)	42.35
	GC content of SSC region (%)	29.23
	GC content of IRB region (%)	42.35
	Overall GC content (%)	36.38

**Table 2:** Summary of assembled gene functions of *E*. *sativa* cp genome.

Category for genes	Groups of genes	Name of genes
Genes involvingin photosynthesis	Subunits of photosystem	*ndhI*, *ndhJ*, *ndhK*, *psaA*, *psaB*, *psaC*, *psaI*, *psaJ*, *psbA*, *psbB*, *psbC*, *psbD*, *psbE*, *psbF*, *psbH*, *psbI*,*psbJ*, *psbK*, *psbL*, *psbM*, *psbN*, *psbT*, *psbZ*
	Subunits of cytochrome b/f complex	*petA*, *petB*^*b*^, *petD*, *petG*, *petL*, *petN*
	Large subunit of Rubisco	*rbcL*
	Subunits of ATP synthase	*atpA*, *atpB*, *atpE*, *atpF*^*b*^, *atpH*, *atpI*
	Subunits of NADH-dehydrogenase	*ndhA*^*b*^, *ndhB*^*a*^^,^^*b*^, *ndhC*, *ndhD*, *ndhE*, *ndhF*, *ndhG*, *ndhH*
Self-replication	Ribosomal RNA genes	*rrn16*^*a*^,*rrn23*^*a*^,*rrn4*.*5*^*a*^,*rrn5*^*a*^
	Transfer RNA genes	*trnA-UGC*^*a*^^,^^*b*^, *trnC-GCA*, *trnD-GUC*, *trnE-UUC*, *trnF-GAA*, *trnfM-CAU*, *trnG-GCC*^*b*^, *trnG-UCC*, *trnH-GUG*, *trnI-CAU*^*a*^, *trnI-GAU*^*a*^^,^^*b*^, *trnK-UUU*^*b*^, *trnL-CAA*^*a*^, *trnL-UAA*^*b*^, *trnL-UAG*, *trnM-CAU*, *trnN-GUU*^*a*^, *trnP-UGG*, *trnQ-UUG*, *trnR-ACG*^*a*^, *trnR-UCU*, *trnS-GCU*, *trnS-GGA*, *trnS-UGA*, *trnT-GGU*, *trnT-UGU*, *trnV-GAC*, *trnV-UAC*^*a*^^,^^*b*^, *trnW-CCA*, *trnY-GUA*
	Small subunit of ribosome	*rps11*, *rps12*^*a*^^,^^*c*^, *rps14*, *rps15*, *rps16*^*b*^, *rps18*, *rps19*, *rps2*
	Large subunit of ribosome	*rps3*, *rps4*, *rps7*^*a*^, *rps8*, *rpl14*, *rpl16*^*b*^, *rpl2*^*a*^^,^^*b*^, *rpl20*, *rpl22*, *rpl23a*,*rpl32*, *rpl33*
	DNA-dependent RNA polymerase	*rpl36*, *rpoA*, *rpoB*, *rpoC1*^*b*^, *rpoC2*
Other genes	Maturase	*matK*
	Envelope membrane protein	*cemA*
	Subunit of acetyl-CoA	*accD*
	C-type cytochrome synthesis gene	*ccsA*
	Protease	*clpP*^*c*^
Functionally unknown genes	Conserved Open reading frames	*ycf1*, *ycf2*^*a*^, *ycf3*^*c*^, *ycf4*, *ycf15*

^a, b, c^ The letters indicate the gene with two copes, harboring one intron and two introns, respectively.

### Long repeat sequence and SSR analysis

**Fig 2:**
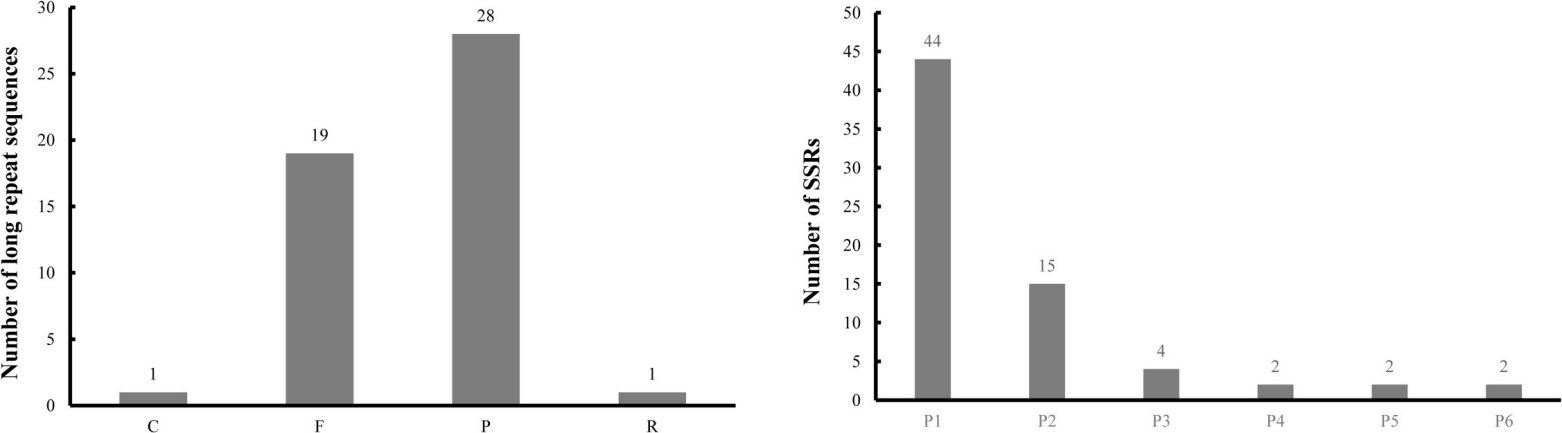
The long repetitive sequences and simple sequence repeats of *E*. *sativa*. (A) The numbers of long repetitive sequences detected in *E*. *sativa* chloroplast genomes, contains four types. F: Forward repeats; R: Reverse repeats; P: Palindrome repeats; C: Complementary. (B) The numbers of SSRs detected in *E*. *sativa* chloroplast genomes, contains six types. P1: mono-; P2: di-; P3: tri-; P4: tetra-; P5: penta-; P6: hexanucleotides.

Long repeat sequences exist widely throughout the genome, and play an essential role in gene expression and regulation. Furthermore, due to the high polymorphism present in these regions, long repeat sequences are ideal for generating genetic and physical maps [39, 40]. In the present study, 49 pairs of long repeat sequences were identified, including 19 forward repeats, 28 palindromic repeats, one reverse repeat (44 bp), and one complementary repetition (49 bp) (Table 3; Fig 2). The longest repeat (85 bp) was a forward type located in the LSC region. Among these long repeats, 30 (61.2%) repeats were found in the LSC region. A majority of the repeat pairs (37 of 49, 75.5%) were found in the same regions, including 30 repeats in the LSC region, five repeats in the IR regions, and two repeats in the SSC region. However, 12 repeats, comprising nine palindromic types and three forward types, were detected in different regions. Complementary repeats are infrequent in other cp genomes in Brassicaceae [41, 42].

**Table 3:** The long repeat sequences detected in *E*. *sativa* cp genome.

No.	Repeat	Type	Repeat 1 Start (bp)	Repeat 2 Start (bp)	Region
1	30	P	61768	61768	LSC
2	30	P	7627	44148	LSC
3	30	F	106858	106890	IRB
4	30	P	106858	129922	IRB; SSC
5	30	P	106890	129954	IRB; SSC
6	32	P	6262	6262	LSC
7	34	F	106854	106886	IRB
8	34	P	106854	129922	IRB; SSC
9	34	P	106886	129954	IRB; SSC
10	34	F	129922	129954	SSC
11	35	P	75818	75818	LSC
12	35	P	64224	64224	LSC
13	36	P	9229	9229	LSC
14	36	P	35536	35536	LSC
15	37	F	97938	119515	IRB; SSC
16	37	P	119515	138867	SSC; IRA
17	39	F	43041	97935	LSC; IRB
18	39	P	43041	138868	LSC; IRA
19	40	P	28188	28188	LSC
20	40	P	75813	75818	LSC
21	40	F	148542	148563	IRA
22	41	P	35531	35536	LSC
23	42	F	97933	119510	IRB; SSC
24	42	P	119510	138867	SSC; IRA
25	44	P	73307	73307	LSC
26	44	R	35505	35505	LSC
27	44	P	76826	76826	LSC
28	44	P	62009	62009	LSC
29	45	P	112907	112907	SSC
30	47	F	88239	88260	IRB
31	47	P	88239	148535	IRB; IRA
32	47	P	88260	148556	IRB; IRA
33	47	F	148535	148556	IRA
34	49	P	29704	29704	LSC
35	49	C	35518	35519	LSC
36	49	P	55354	55354	LSC
37	50	P	185	185	LSC
38	52	F	37938	40162	LSC
39	55	F	37935	40159	LSC
40	55	P	66074	66074	LSC
41	56	P	179	185	LSC
42	58	F	37932	40156	LSC
43	73	F	37917	40141	LSC
44	74	F	37899	40123	LSC
45	76	F	37914	40138	LSC
46	77	F	37896	40120	LSC
47	81	F	37909	40133	LSC
48	82	F	37902	40126	LSC
49	85	F	37905	40129	LSC

Note: P represents for palindrome, R for reverse, F for forward, and C for complement types.

SSR loci are widely found in various species and are useful for studies of molecular evolution and genetic diversity as well as the development of molecular markers essential for plant breeding [43, 44]. Sixty-nine SSRs were identified in the *E*. *sativa* cp genome (Table 4; Fig 2), including 44 mononucleotides, 15 dinucleotides, four trinucleotides, two tetranucleotides, two pentanucleotides, and two hexanucleotides with a length of at least 10 bp. Fewer SSR loci were detected than the number of SSR loci reported in other cp genomes of Brassicaceae [16, 18, 41, 45]. Among the 44 mononucleotide repeats (including 15 A type, 27 T type, one G type, and one C type), the longest SSR was one T type of 17 bp, which was found in the SSC regions. Similar distributions of mononucleotide repeats were observed in the cp genomes of *B*. *napus* [45], *Raphanus sativus* [18], *Nasturium officinale* [41], and *Sinapis alba* [16]; however, the mononucleotide repeats of the G type was only observed in this cp genome. The AT/TA type contributed to all 14 dinucleotides, and the longest type of dinucleotides was AT type of 20 bp. Four trinucleotide repeats were detected, including two AAT (12 bp) types and two ATT (12 bp), which were located in the LSC and IR regions respectively. Two tetranucleotides repeats, CAAA (12 bp) and ATAG (12 bp), were found in the LSC and SSC regions, respectively. Two pentanucleotides (TGTTG and CAACA) and two hexanucleotides (GAAAGT and GTTAGA) were also detected in this cp genome. The number and types of SSR loci varied extensively compared to other cp genomes in Brassicacea using the same identified software and criteria, which supports the idea that these SSRs can be made into lineage-specific markers for genetic diversity analysis. Furthermore, SSRs have been used as markers to understand the evolutionary history [46, 47].

**Table 4:** Distribution of SSRs in the *E*. *sativa* cp genome.

SSR Type	Unit	Length	Number	Position on Genome (bp)
P1	A	10	9	4258–4267,12905–12914,26968–26998,34531–34540,55461–55470,70959–70968,96292–96301,107217–107226,109509–109518
		11	3	8250–8260,67289–67299,137563
		12	2	65299–65310,113869–113880
		14	1	64397–64310
	T	10	12	25276–25285,42704–42713,44425–44434,55900–55909,59298–59307,70238–70247,80704–80713,81340–81349,123978–123987,127325–127334,129617–129626,140542–140551
		11	5	17535–17545,63532–63542,99270–99280,125451–125461,126154–126164
		12	2	69411–69422,111972–111983
		13	4	41304–41316,77182–77194,81603–81615,81736–81748
		15	2	49040–49054,78519–78533
		16	1	124947–124962
		17	1	114766–114782
	C	10	1	47447–47456
	G	10	1	66124–66133
P2	AT	10	6	13357–13366,833116–83125,107641–107650,120486–120495,129193–129202,143196–143205
		16	1	30693–30708
		18	1	35541–35558
		20	1	3718–3737
	TA	10	5	6274–6283,18907–18916,93637–93646,111792–111801,122991–123000
		12	1	7831–7842
P3	AAT	12	2	12649–12660,89584–89595
	ATT	12	2	45840–45851,147248–147259
P4	CAAA	12	1	28035–28046
	ATAG	12	1	111554–111565
P5	TGTTG	15	1	98769–98783
	CAACA	15	1	138060–138074
P6	GAAAGT	18	1	56545–56562
	GTTAGA	18	1	80995–81012

### Codon biased usage analysis

**Fig 3:**
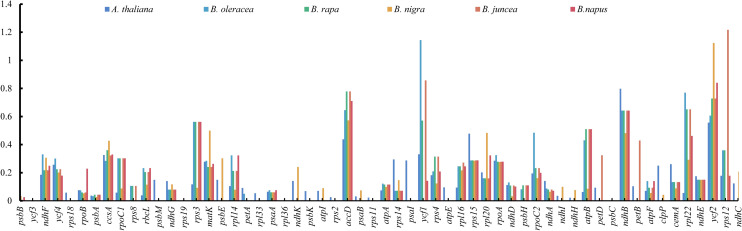
Codon content of 20 amino acids and stop codons in all protein-coding genes of the *E*. *sativa* chloroplast genome. Those whose RSCU value is greater than 1 are bold by the font.

**Fig 4:**
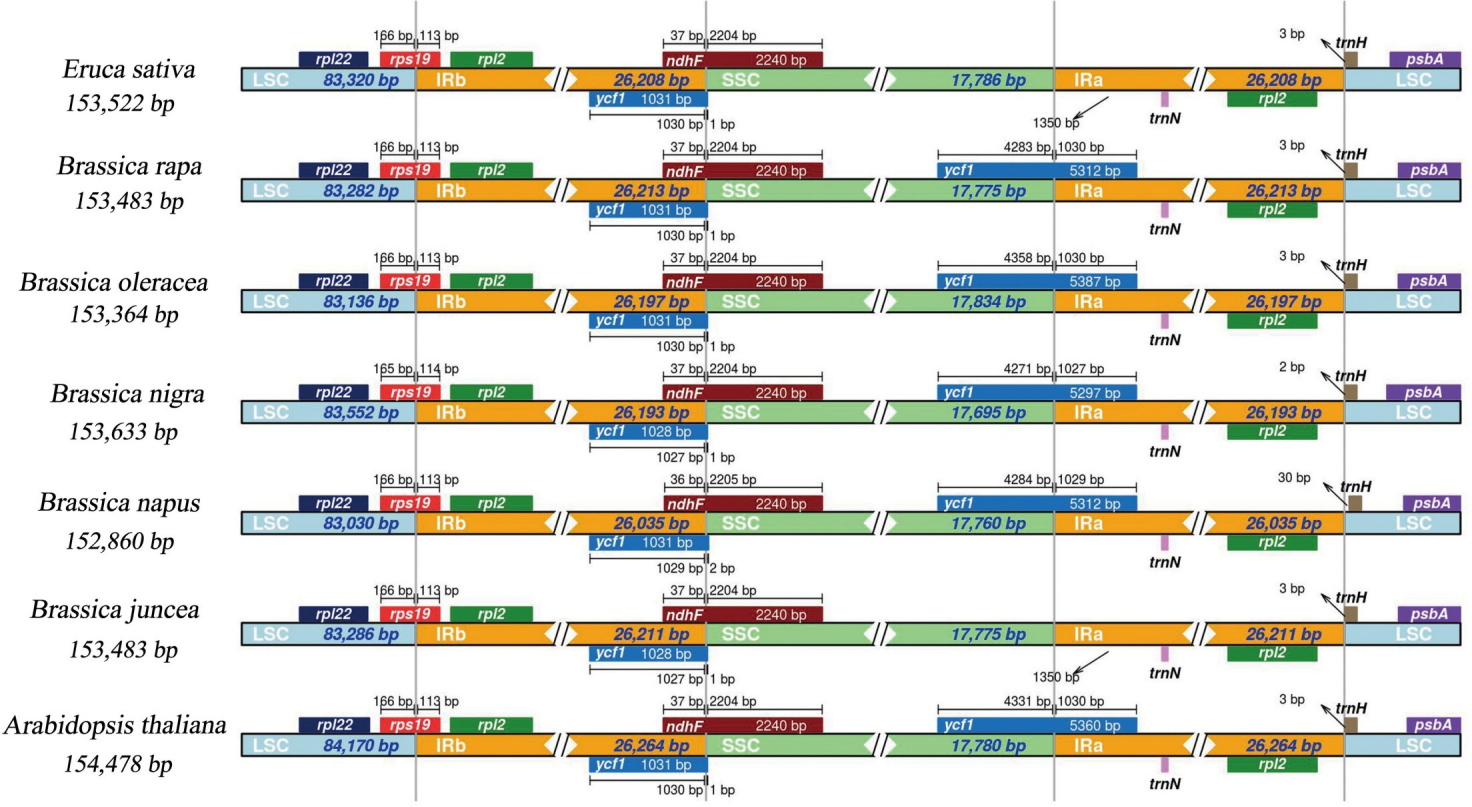
Comparison of the borders of the LSC, SSC, and IR regions in cp genomes of six species of Brassicaceae.

Codon usage bias exists widely in plastoms and is believed to play a key role in reshaping the cp genome [48]. Moreover, the codon usage bias of some genes in plastoms likely responds to outside pressure [49]. In this study, the codon usage bias and RSCU were analyzed based on 85 CDs (coding sequences) sequences in the *E*. *sativa* cp genome. These CDs generated a sequence of 74,547 bp in length, which encoded 24,849 amino acids (Table 5). Of these acids, 2,658 (10.70%) were leucine, representing the most popular type, followed by isoleucine (2,134 codons, 8.59%) and serine (1915 codons, 7.71%), whereas only 300 (1.21%) cysteines were detected (Fig 3). The detailed information of codon usage of the 85 CDs in the *E*. *sativa* cp genome is listed in Fig 4. The RSCU values of 29 codons were >1, indicating that these codons were preferentially used. Among these biased codons, the codon for leucine (UUA), was the most preferred codon with an RSCU value of 2.03. The UGG (tryptophan) and AUG (methionine) codons showed no biased usage (RSCU = 1). All of the biased codons ended with A/U, except UUG, which agrees with the results for the *N*. *officinale* [41] and *S*. *alba* cp genomes [16], suggesting that codon usage of the cp genome in Brassicaceae is conserved.

**Table 5:** Summary of codon usage and amino acids patterns of *E*. *sativa* cp genome.

Codon	Number	Amino acids	Ratio of Codon	RSCU	Number of amino acid	Ratio of amino acid
GCA	369	Ala	1.48%	1.09	1348	5.42%
GCC	207		0.83%	0.61		
GCG	146		0.59%	0.43		
GCU	626		2.52%	1.86		
AGA	425	Arg	1.71%	1.72	1482	5.96%
AGG	155		0.62%	0.63		
CGA	345		1.39%	1.40		
CGC	106		0.43%	0.29		
CGG	118		0.47%	0.32		
CGU	333		1.34%	0.90		
AAC	271	Asn	1.09%	0.46	1180	4.75%
AAU	909		3.66%	1.54		
GAC	190	Asp	0.76%	0.39	987	3.97%
GAU	797		3.21%	1.61		
UGC	73	Cys	0.29%	0.49	300	1.21%
UGU	227		0.91%	1.51		
CAA	680	Gln	2.74%	1.54	881	3.55%
CAG	201		0.81%	0.46		
GAA	960	Glu	3.86%	1.51	1271	5.11%
GAG	311		1.25%	0.49		
GGA	709	Gly	2.85%	1.66	1712	6.89%
GGC	158		0.64%	0.37		
GGG	285		1.15%	0.67		
GGU	560		2.25%	1.31		
CAC	145	His	0.58%	0.50	577	2.32%
CAU	432		1.74%	1.50		
AUA	663	Ile	2.67%	0.93	2134	8.59%
AUC	407		1.64%	0.57		
AUU	1064		4.28%	1.50		
CUA	356	Leu	1.43%	0.80	2658	10.70%
CUC	173		0.70%	0.39		
CUG	166		0.67%	0.37		
CUU	554		2.23%	1.25		
UUA	900		3.62%	2.03		
UUG	509		2.05%	1.15		
AAA	962	Lys	3.87%	1.49	1291	5.20%
AAG	329		1.32%	0.51		
AUG	561	Met	2.26%	1.00	561	2.26%
UUC	482	Phe	1.94%	0.66	1466	5.90%
UUU	984		3.96%	1.34		
CCA	291	Pro	1.17%	1.11	1017	4.09%
CCC	189		0.76%	0.72		
CCG	135		0.54%	0.52		
CCU	402		1.62%	1.54		
AGC	123	Ser	0.49%	0.39	1915	7.71%
AGU	388		1.56%	1.22		
UCA	374		1.51%	1.17		
UCC	284		1.14%	0.89		
UCG	191		0.77%	0.60		
UCU	555		2.23%	1.74		
UAA	51	TER	0.21%	1.78	86	0.35%
UAG	22		0.09%	0.77		
UGA	13		0.05%	0.45		
ACA	392	Thr	1.58%	1.22	1282	5.16%
ACC	225		0.91%	0.70		
ACG	143		0.58%	0.45		
ACU	522		2.10%	1.63		
UGG	419	Trp	1.69%	1.00	419	1.69%
UAC	172	Tyr	0.69%	0.37	919	3.70%
UAU	747		3.01%	1.63		
GUA	498	Val	2.00%	1.46	1363	5.49%
GUC	168		0.68%	0.49		
GUG	195		0.78%	0.57		
GUU	502		2.02%	1.47		

### Comparative analysis of cp genomes of six related species

To detect divergence between the *E*. *sativa* cp genome and its related species, six reported cp genome sequences in Brassicaceae were downloaded from the NCBI, including five important *Brassica* species (*B*. *rapa*, *B*. *oleracea*, *B*. *juncea*, *B*. *nigra*, and *B*. *napus*), and the model species: *A*. *thaliana*. As shown in Table 6, these cp genomes were generally highly conserved. Briefly, the sequences ranged from 152,860 bp (*B*. *napus*) to 154,478 bp (*A*. *thaliana*) in length, and each component of the quadripartite cycle was comparable among the selected cp genomes. The overall GC content was also quite similar (36.29–36.39%) among these cp genomes. The gene content in these cp genomes was consistent, except that the *ycf15* gene was missing in the *A*. *thaliana* cp genome. The *ycf15* gene was also missing in the *S*. *alba* cp genome [16], indicating that this gene was varied widely in Brassicaceae. The adjacent genes and boundaries of LSC/IRb/SSC/IRa among the seven related cp genomes were compared (Fig 4), because the variable boundary regions that are believed to be adriving force for the variation in the angiosperm cp genomes [50]. In this study, the IRb/LSC boundary was located within the coding region of the *rps19* gene in all seven selected species. Furthermore, the *rps19* gene had an expansion of 113/114 bp in the IRb region in all selected cp genomes. The *trnH*-GUG gene and *rpl2* gene resided at the LSC/IRa border, respectively, in all seven cp genomes, and the *trnH* gene was 2–30 bp from the border. Additionally, the boundary of IRb/SSC was located in the repetitive regions of the *ycf1* and *ndhF* genes in all seven species, with only 1 bp of *ycf1* and 36 bp of *ndhF* located in the SSC region. The IRa/SSC junction extended into the *ycf1* gene in the other five species and the length of the *ycf1* genes ranged from 1,027 bp to 1,030 bp in the IRa region, but the *ycf1* gene was missing from the IRb/SSC regions in the *E*. *sativa* and *B*. *juncea* cp genomes. Similar results were observed in mustard species, such as *S*. *alba* [16] and *S*. *arvensis*.

**Table 6:** Comparison analyses of cp genomes among seven Brassicaceae species.

Genome Features	*E*. *sativa*	*A*. *thaliana*	*B*. *juncea*	*B*. *napus*	*B*. *nigra*	*B*. *rapa*	*B*. *oleracea*
Genome Size (bp)	153522	154478	153483	152860	153633	153482	153364
LSC length (bp)	83320	84170	83268	83030	83552	83282	83136
SSC length (bp)	17786	17780	17775	17760	17695	17775	17834
IR length (bp)	26208	26264	26211	26035	26193	26213	26197
GC content (%)	36.38	36.29	36.36	36.32	36.39	36.36	36.36
Genome number	113	113	113	113	114	113	114
Protein-coding gene	79	79	79	79	80	79	80
rRNA	4	4	4	4	4	4	4
tRNA	30	30	30	30	30	30	30

**Fig 5:**
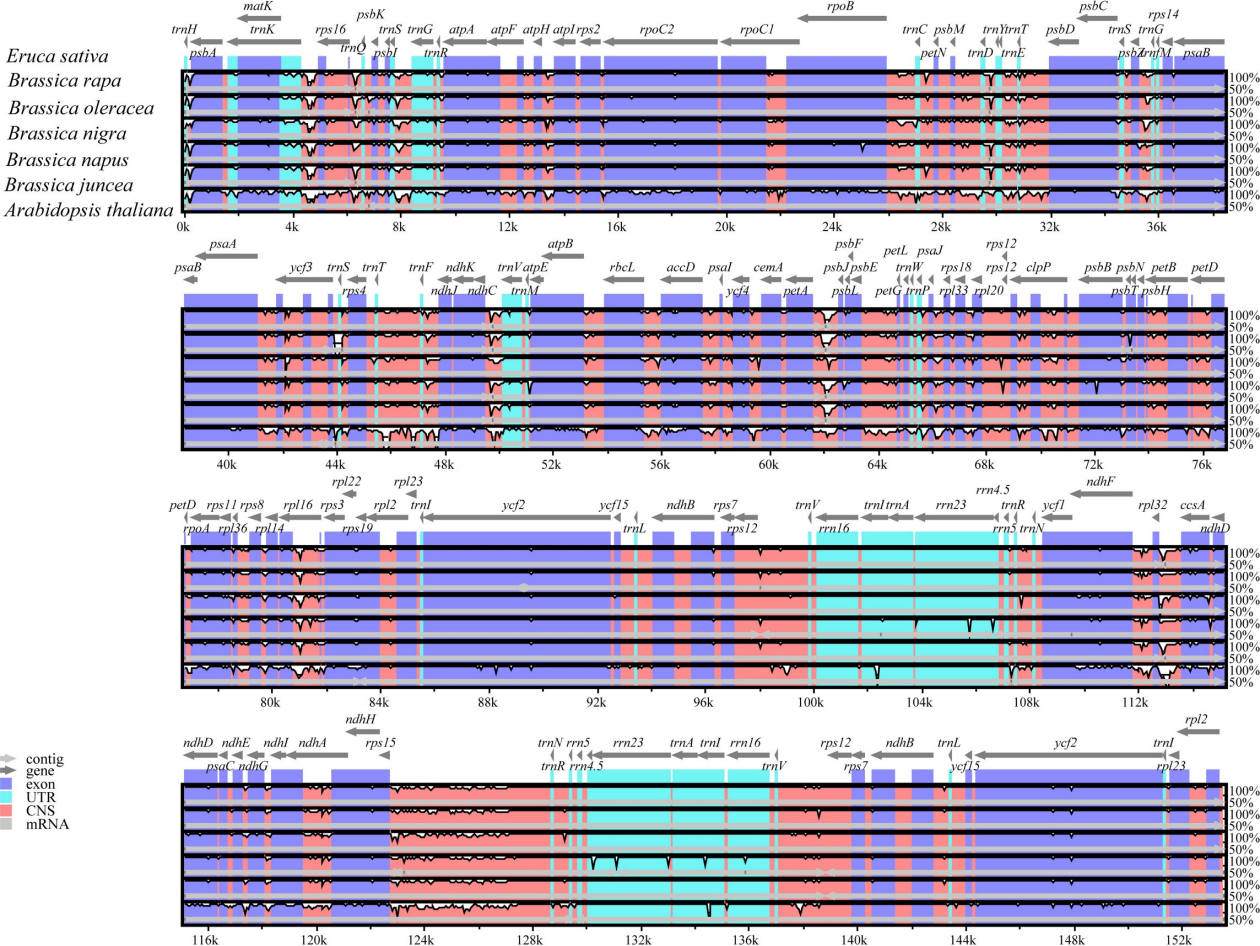
Sequence alignment of seven cp genomes of Brassicaceae by mVISTA, with the annotation of *E*. *sativa* as the reference. The vertical scale indicates the percentage of identity, ranging from 50% to 100%. The horizontal axis indicates the coordinates within the chloroplast genome. Genome regions are color coded as protein-coding, rRNA, tRNA, intron, and conserved noncoding sequences.

**Fig 6:**
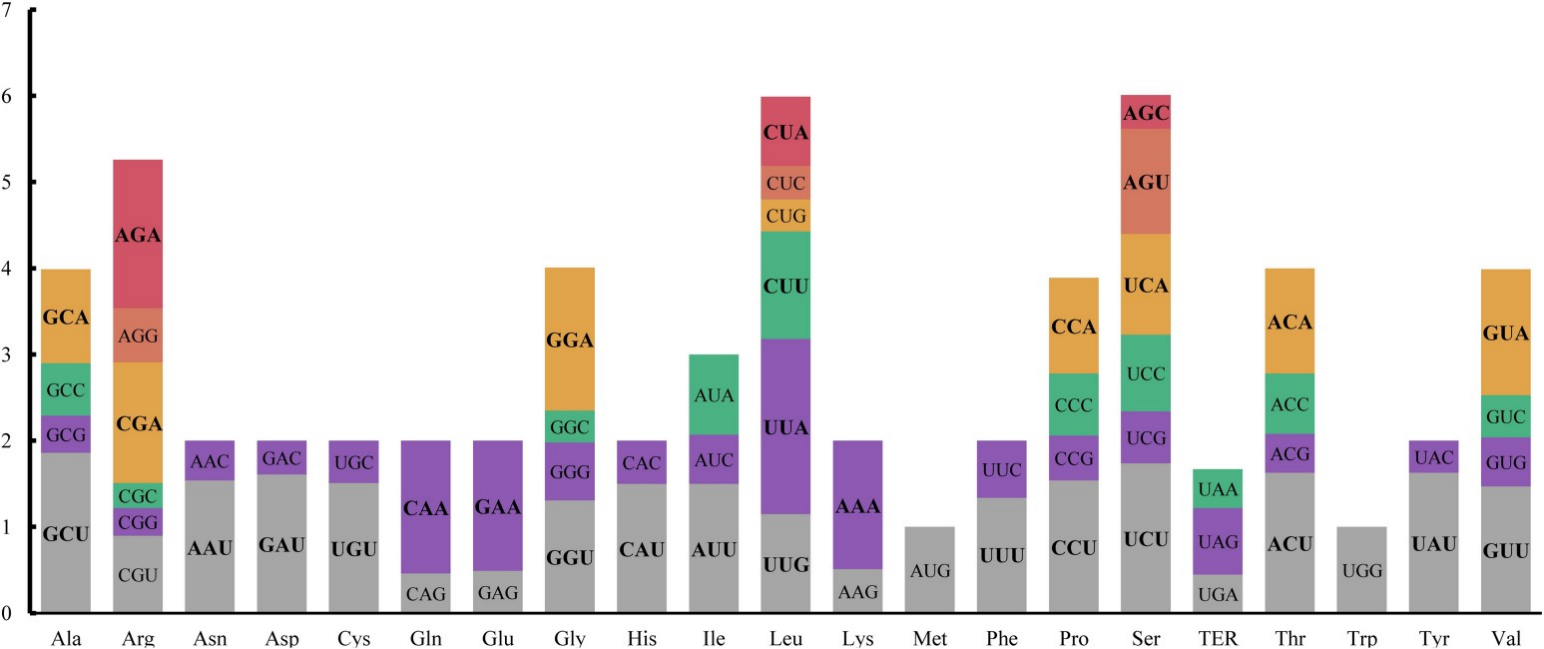
The Ka/Ks ratios of 79 protein-coding genes of the *E*. *sativa* cp genome versus six closely related species of Brassicaceae.

To further detect divergence of the cp genomes among related species, and to verify whether genome rearrangement had taken place in the *E*. *sativa* cp genome, we used mVISTA to compare the homology of the whole cp sequence among the seven selected cp genomes of Brassicaceae, using the *E*. *sativa* cp genome as a reference (Fig 5). The results showed that no genome structural rearrangement had occurred in any of the selected cp genomes, and the selected cp genomes were highly conserved with a genome similarity of > 90%. However, the non-coding regions were more divergent than the coding regions, and the LSC and SSC regions were also more divergent than the IR regions. Furthermore, the *matk*, *atpA*, *rpoC2*, *accD*, *rpoA*, *rps19*, *ycf2*, *ycf1* and *ccsA* genes were quite mutable. Synonymous (Ks) and non-synonymous (Ka) nucleotide substitution patterns of genes are important indicators of gene evolution [18]. The Ka/Ks ratio is used to assess whether there are selective pressures on protein-coding genes or to evaluate the rate of gene divergence. Ka/Ks ratios > 1, close to 1, or < 1 indicate that the gene has undergone positive selection, neutral selection, or purifying selection, respectively [51]. In this study, we calculated the Ka/Ks ratios of the *E*. *sativa* cp genome compared to six closely related species, including *B*. *rapa*, *B*. *oleracea*, *B*. *juncea*, *B*. *nigra*, *B*. *napus* and *A*. *thaliana* (Fig 6). A total of 79 homologous CDs were selected to calculate the Ka/Ks among the selected cp genomes. The results showed that the average Ka/Ks ratio was 0.17, after removing the genes with Ka or Ks of 0, indicating that the genes in the *E*. *sativa* cp genome were subject to strong purifying selection pressures. The majority of genes had a Ka/Ks ratio < 0.5 in all comparisons, and the Ka/Ks ratios of most of the genes were comparable among each of the comparisons, except the *rps3*, *accD*, *ndhB*, *rpl22*, *ycf1*, *ycf2*, and *rps12* genes of which the Ka/Ks ratio was elusive. For example, the Ka/Ks ratio of *ycf1* was 1.144 in the comparison of *B*. *oleracea* vs. *E*. *sativa*, but the ratio was < 1 in the other five comparisons. Similar results were observed for *ycf2* and *rpl22*. The *ycf1* and *ycf2* genes are considered to be pseudogenes and have been believed to have no function in plants for a long time [52, 53]. However, knockout studies have shown that the *ycf1* gene is indispensable for plant cell survival [45]. The various Ka/Ks ratios of the *ycf2* gene observed in the *S*. *alba* cp genome compared to other related species, indicate that the *ycf2* gene was reshaped in response to outside selection stress. It is the largest plastid gene reported in angiosperms, but the function of *ycf2* is largely unknown [54].

### Phylogenetic analysis of the Brassicaceae

**Fig 7:**
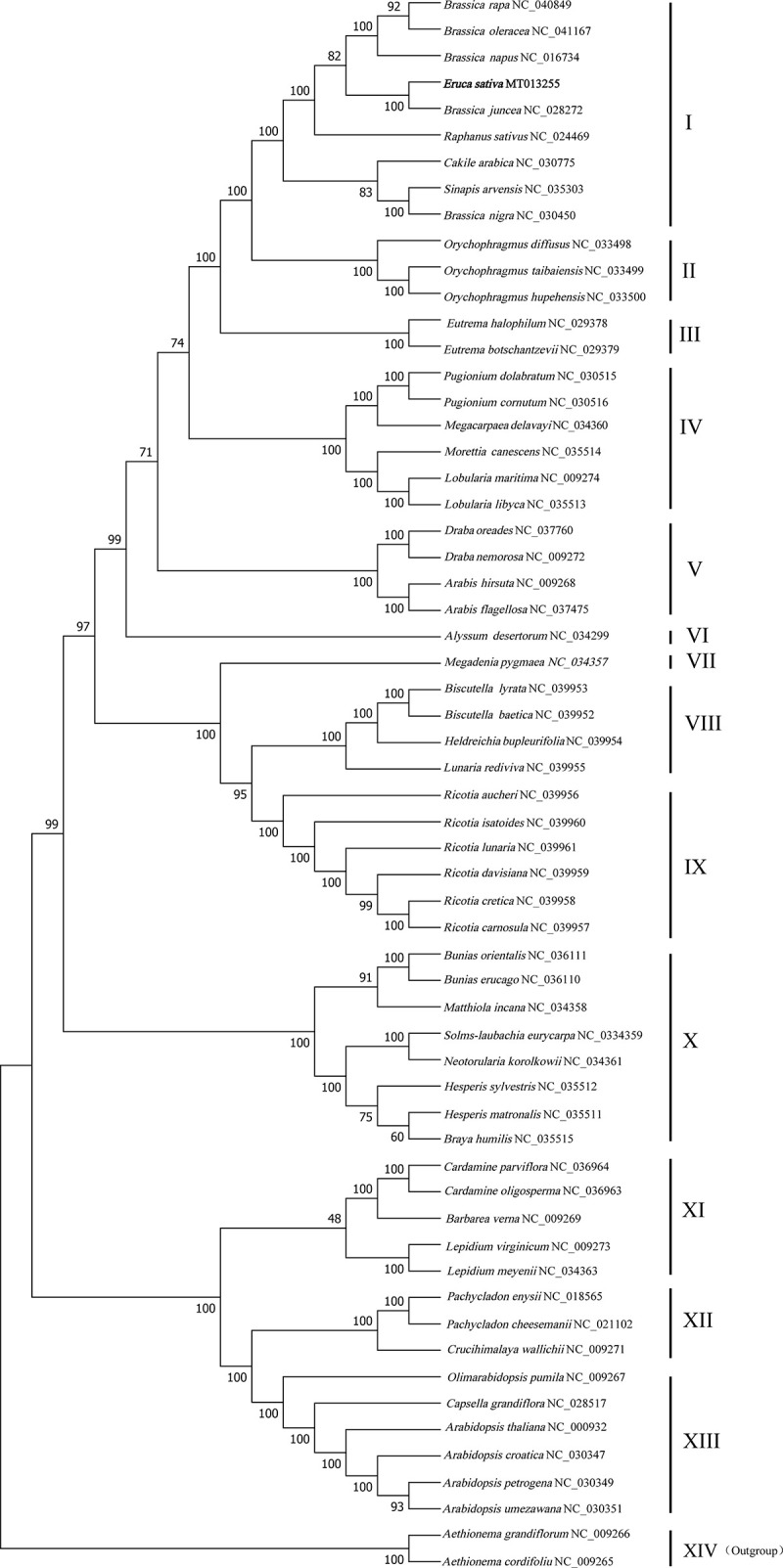
Phylogenetic relationships of the 59 species of Brassicaceae constructed from the shared protein-coding gene sequences using Maximum Likelihood (ML). **The** *Aethionema* genus formed the outgroup.

Cp genomes containing a large amount of genetic information have been more accessible with the development of high-throughput sequencing technology. Accordingly, several studies have employed cp genomes to detect the phylogenetic relationships in the Brassicaceae family [15, 16, 18, 41]. Although several studies have systematically elucidated the phylogenetic relationships of Brassicaceae species based on nuclear gene [55–57] and cp gene information [16, 41, 58], the systematic position of *E*. *sativa* remains unclear. Based on the *AG* (cis-regulatory sequences of the floral homeotic gene) gene, *E*. *sativa* is closely related to *S*. *alba* [59]. Another study employing several cp genes to construct the phylogenetic relationship demonstrated that the genus *Eruca* had the closest relationship with *Diplotaxis harra* [58]. However, both studies indicated that the genus *Eruca* is closely related to the genus *Brassica*. In this study, the cp genomes of 59 Brassicaceae species were used for the phylogenetic analysis based on 62 homologous CDs. The phylogenetic tree generated 58 branches with node values > 48% (Fig 7). Among these branches, 50 branches had the node values > 90%. Based on the node values, the branches were divided into 14 subclades, and *E*. *sativa*, *R*. *sativus*, *Cakile arabica*, *S*. *arvensis*, and *Brassica* species were classified into the same subclade. Intriguingly, *E*. *sativa* and *B*. *juncea* constructed a single branch supported by a node value of 100%, indicating that *E*. *sativa* s was most closely related to *B*. *juncea*. Another study demonstrated that *E*. *sativa* is closed related to the genus *Brassica* based on homology of the mitochondrial genome [60]. Furthermore, *B*. *nigra* was most closely related to *S*. *arvensis*, in line with the previous result that *B*. *nigra* is closer to the genus *Sinapsis* than other *Brassica* species at the cp genomic level [16]. These studies support that the genera *Brasscia*, *Eruca*, *Sinapsis*, and *Raphanus* share a similar ancestor, or exchanges/captures of maternal genetic information occurred among these species during speciation.

## Conclusions

In the present study, we obtained the complete cp genome of *E*. *sativa* using the combination of PacBio Sequel and Illumina HiSeq reads. The results showed that the cp genome had a typical quadripartite structure of 153,522 bp, consisting of two copies of inverted repeat (IRa and IRb) regions of 26,208 bp separated by one LSC region of 83,320 bp and one SSC region of 17,786 bp. The cp genome harbored 112 unique genes, including 79 protein-coding genes, 29 tRNA genes, and four rRNA genes. The synonymous (Ks) and non-synonymous (Ks) substitution rate analysis showed that protein-coding genes generally underwent purifying selection pressure, except *ycf1* and *ycf2*. A phylogenetic analysis revealed that *E*. *sativa* is closely related to agriculturally important *Brassica* species, and most closely related to *B*. *juncea*, indicating that it may be possible to transfer favorable *E*. *sativa* alleles into other *Brassica* species. These results are helpful to further genetic improvement and breeding of *E*. *sativa*, and also provide valuable information for understanding the evolutionary history of *E*. *sativa*.

## Supporting information

S1 Table List of the cp genome of 59 Brassicaceae species used for phylogenetic analysis. (DOCX)

S2 Table Summary of de novo sequencing of cp genome of E. sativa. (DOCX)

## Abbreviations

Cp: chloroplast
IR: inverted repeat
LSC: large single copy
SSC: small single copy
rRNA: ribosomal RNA
tRNA: transfer RNA
SSR: simple sequence repeat
RSCU: the relative synonymous codon usage
ML: maximum likelihood
Ka: non-synonymous
Ks: synonymous
