# Supplementary material for: Complete chloroplast genome features and phylogenetic analysis of Eruca sativa (Brassicaceae)
Source: PLoS One. 2021 Mar 12;16(3):e0248556. doi: 10.1371/journal.pone.0248556 (PMC7954331; doi:10.1371/journal.pone.0248556)
Supplement: S1 Table — (DOCX) [file pone.0248556.s001.docx]

**S1 Table. List of the cp genome of 59 Brassicaceae species used for phylogenetic analysis**

| Latin Name | Genus | NCBI Accession |
| --- | --- | --- |
| *Aethionema cordifolium* | *Aethionema* | NC_009265 |
| *Aethionema grandiflorum* | *Aethionema* | NC_009266 |
| *Alyssum desertorum* | *Alyssum* | NC_034299 |
| *Arabidopsis croatica* | *Arabidopsis* | NC_030347 |
| *Arabidopsis petrogena* | *Arabidopsis* | NC_030349 |
| *Arabidopsis thaliana* | *Arabidopsis* | NC_000932 |
| *Arabidopsis umezawana* | *Arabidopsis* | NC_030351 |
| *Arabis flagellosa* | *Arabis* | NC_037475 |
| *Arabis hirsuta* | *Arabis* | NC_009268 |
| *Barbarea verna* | *Barbarea* | NC_009269 |
| *Biscutella baetica* | *Biscutella* | NC_039952 |
| *Biscutella lyrata* | *Biscutella* | NC_039953 |
| *Brassica juncea* | *Brassica* | NC_028272 |
| *Brassica napus* | *Brassica* | NC_016734 |
| *Brassica nigra* | *Brassica* | NC_030450 |
| *Brassica oleracea* | *Brassica* | NC_041167 |
| *Brassica rapa* | *Brassica* | NC_040849 |
| *Braya humilis* | *Braya* | NC_035515 |
| *Bunias erucago* | *Bunias* | NC_036110 |
| *Bunias orientalis* | *Bunias* | NC_036111 |
| *Cakile arabica* | *Cakile* | NC_030775 |
| *Capsella grandiflora* | *Capsella* | NC_028517 |
| *Cardamine oligosperma* | *Cardamine* | NC_036963 |
| *Cardamine parviflora* | *Cardamine* | NC_036964 |
| *Crucihimalaya wallichii* | *Crucihimalaya* | NC_009271 |
| *Draba nemorosa* | *Draba* | NC_009272 |
| *Draba oreades* | *Draba* | NC_037760 |
| *Eutrema botschantzevii* | *Eutrema* | NC_029379 |
| *Eutrema halophilum* | *Eutrema* | NC_029378 |
| *Heldreichia bupleurifolia* | *Heldreichia* | NC_039954 |
| *Hesperis matronalis* | *Hesperis* | NC_035511 |
| *Hesperis sylvestris* | *Hesperis* | NC_035512 |
| *Lepidium meyenii* | *Lepidium* | NC_034363 |
| *Lepidium virginicum* | *Lepidium* | NC_009273 |
| *Lobularia libyca* | *Lobularia* | NC_035513 |
| *Lobularia maritima* | *Lobularia* | NC_009274 |
| *Lunaria rediviva* | *Lunaria* | NC_039955 |
| *Matthiola incana* | *Matthiola* | NC_034358 |
| *Megacarpaea delavayi* | *Megacarpaea* | NC_034360 |
| *Megadenia pygmaea* | *Megadenia* | NC_034357 |
| *Morettia canescens* | *Morettia* | NC_035514 |
| *Neotorularia korolkowii* | *Neotorularia* | NC_034361 |
| *Olimarabidopsis pumila* | *Olimarabidopsis* | NC_009267 |
| *Orychophragmus hupehensis* | *Orychophragmus* | NC_033500 |
| *Orychophragmus taibaiensis* | *Orychophragmus* | NC_033499 |
| *Orychophragmus diffusus* | *Orychophragmus* | NC_033498 |
| *Pachycladon cheesemanii* | *Pachycladon* | NC_021102 |
| *Pachycladon enysii* | *Pachycladon* | NC_018565 |
| *Pugionium cornutum* | *Pugionium* | NC_030516 |
| *Pugionium dolabratum* | *Pugionium* | NC_030515 |
| *Raphanus sativus* | *Raphanus* | NC_024469 |
| *Ricotia aucheri* | *Ricotia* | NC_039956 |
| *Ricotia carnosula* | *Ricotia* | NC_039957 |
| *Ricotia cretica* | *Ricotia* | NC_039958 |
| *Ricotia davisiana* | *Ricotia* | NC_039959 |
| *Ricotia isatoides* | *Ricotia* | NC_039960 |
| *Ricotia lunaria* | *Ricotia* | NC_039961 |
| *Sinapis arvensis* | *Sinapis* | NC_035303 |
| *Solms-laubachia eurycarpa* | *Solms-laubachia* | NC_034359 |
