# Supplementary material for: Complete chloroplast genome features and phylogenetic analysis of Eruca sativa (Brassicaceae)
Source: PLoS One. 2021 Mar 12;16(3):e0248556. doi: 10.1371/journal.pone.0248556 (PMC7954331; doi:10.1371/journal.pone.0248556)
Supplement: S2 Table — (DOCX) [file pone.0248556.s002.docx]

**S2 Table. Summary of *de novo* sequencing of cp genome of *E. sativa***

| Category | Items | Descriptions |
| --- | --- | --- |
| PacBio Sequel Platform | Library Size (kb) | 20 |
|  | Subreads Number | 20,921 |
|  | Subreads Bases (bp) | 83,717,860 |
|  | Subreads N50 Length(bp) | 4,698 |
|  | Subreads N90 Length(bp) | 2,063 |
|  | Subreads Average Length(bp) | 4,002 |
| Illumina HiSeq Platform | Library Size (bp) | 450 |
|  | Raw data (Mb) | 8,078 |
|  | Clean data (Mb) | 7,722 |
|  | Clean data Q20(%) | 97.62 |
